# COVID-19 Screening for Healthcare Workers in a Tertiary Infectious Diseases Referral Hospital in Manila, the Philippines

**DOI:** 10.4269/ajtmh.20-0715

**Published:** 2020-07-29

**Authors:** Annavi Marie G. Villanueva, Jezreel Lazaro, Ana Ria Sayo, Su Myat Han, Tatsuya Ukawa, Shuichi Suzuki, Saho Takaya, Elizabeth Telan, Rontgene Solante, Koya Ariyoshi, Chris Smith

**Affiliations:** 1 San Lazaro Hospital – Nagasaki University Collaborative Research Office, Manila, Philippines;; 2 School of Tropical Medicine and Global Health, Nagasaki University, Nagasaki, Japan;; 3 San Lazaro Hospital, Manila, Philippines;; 4 Faculty of Infectious and Tropical Diseases, London School of Hygiene and Tropical Medicine, London, England;; 5 Institute of Tropical Medicine, Nagasaki University, Nagasaki, Japan

## Abstract

COVID-19 is an emerging disease threatening the lives of patients and healthcare workers (HCWs) alike. In this article, we present initial results of COVID-19 screening performed among the hospital staff of an infectious diseases referral hospital in Manila, the Philippines. Of 324 HCWs tested, eight were positive; only one was exposed to COVID-19 patients, whereas seven others belonged to two different departments. Routine screening of hospital staff is invaluable for the safety of the HCWs and the patients in hospitals and should be performed on a regular basis. In monitoring HCWs, we protect one of our most valuable assets against COVID-19.

## INTRODUCTION

COVID-19, caused by SARS-CoV-2, has spread to almost every region and country of the world. Frontline healthcare workers (HCWs) are at particular risk. A large number of HCWs have already been infected, and some of them had already lost their lives. The International Council of Nurses estimated that more than 90,000 HCWs had been infected worldwide.^1^ In the Philippines, there were already 2,710 COVID-19–confirmed cases among HCWs as of June 6, 2020.^2^ Protecting hospital frontline staff is vital to sustain the operation of the hospital and provision of health care to patients. In this article, we describe our experience of screening HCWs in San Lazaro Hospital (SLH), a tertiary referral hospital for infectious diseases in Manila, the Philippines, where the first two COVID-19 cases in the Philippines were confirmed on January 31, 2020 and February 1, 2020, respectively.^3^

The first laboratory-confirmed COVID-19 case of a HCW in SLH was reported on March 20, 2020. This led to great anxiety and fear among HCWs in the hospital. At this time, however, COVID-19 diagnostic testing was only available at the Research Institute for Tropical Medicine, the national reference laboratory, for individuals fulfilling the following criteria^1^: presence of fever and/or cough, shortness of breath or other respiratory symptoms, and^2^ travel or residence in an area with local transmission of COVID-19, or close contact with a confirmed COVID-19 case. Close contact was defined as providing direct care without appropriate personal protective equipment (PPE), staying in the same close environment, and traveling together in close proximity (1 m) in any kind of conveyance.^4^

To support hospital operations with infection control measures against further spread, COVID-19 screening for hospital staff was initiated at the SLH-Nagasaki University Collaborative Research Laboratory (SLH-NU) on the same day that the first HCW case was detected. Screening activities were implemented under an existing epidemiological research study on COVID-19 in SLH with ethical approval from the SLH research ethics and review unit (Ref: SLH-RERU-2020-022-I) and the School of Tropical Medicine and Global Health, Nagasaki University Ethical Committee (NU_TMGH_2020_119_1).

## MECHANISMS OF SCREENING

Screening activities were prioritized among HCWs who were in close contact with the first COVID-positive case. Subsequently, a clinic responsible for the hospital staff health was designated to identify HCWs for screening. Hospital staff with known COVID-19 exposure or related signs and symptoms received consultation at the clinic and were referred to the SLH-NU for testing. The criteria for referral for testing were as follows: 1) history of close contact or high-risk exposure with a confirmed COVID-19 case as defined by the WHO Global Surveillance for COVID-19^5^ or 2) development of COVID-related signs and symptoms.

Risk assessment was performed according to the CDC guideline.^6^ High-risk exposure was defined as 1) prolonged close contact of a HCW with a COVID-19 patient while both the HCW and patient were without face masks; or 2) same-room exposure to aerosolizing procedures on a COVID-19 patient while the HCW was without a face mask or goggles. Medium-risk exposure was defined as 3) prolonged close contact with a COVID-19 patient with face mask while the HCW was not wearing a mask or goggles; or 4) prolonged close contact with a patient while the HCW was wearing a gown, gloves, eye protection, and a face mask (versus respirator) during an aerosol generating procedure. Low-risk exposure was defined as 5) brief interactions with a COVID-19 patient or 6) prolonged close contact with a patient wearing a cloth mask/face mask while the HCW was wearing a face mask or respirator.

Both nasopharyngeal and oropharyngeal swab specimens were obtained using HydraFlock Sterile Flocked Collection Devices (Puritan^®^, Guilford, ME). Viral RNA was extracted and purified from the specimen using QIAamp^®^ Viral RNA Mini Kit (Qiagen, Hilden, Germany). The RNA extracts were tested using real-time reverse transcriptase PCR (rRT-PCR) for the presence of SARS-CoV-2 viral RNA using primers and probes from Corman et al.^7^ and Nao et al.^8^ protocols. Synthesized oligonucleotides were used as positive controls. Primers and probes to detect *RnaseP* were used as internal control. In each run, a no-template control containing only the master mix with PCR-grade water was added. Samples for rRT-PCR were placed in single tubes. Viral RNA extraction, reagent preparation for rRT-PCR, and addition of template RNA were performed in separate rooms using previously decontaminated and ultraviolet-irradiated class II type A2 biosafety cabinets.

We summarized demographic characteristics, comorbidities, and signs and symptoms by whether screened cases tested positive or not. Continuous variables were expressed as mean (SD) and median (range), and categorical variables were expressed as number (%). Fisher’s exact test was used to test for associations between categorical variables, and the Mann–Whitney test was used to compare discrete variables between categories of categorical variables. All analyses were conducted using Stata v. 15 (StataCorp LLC, College Station, TX).

## RESULTS

From March 20 to April 20, 324 COVID-19 PCR tests were performed from SLH’s 1,213 HCW staff (141 doctors, 299 nurses, 198 nursing aides, 90 clinical staff, and 485 nonclinical employees and administrators) at the SLH-NU laboratory (see Supplemental Table 1). Of 324 tests, 97 were categorized as moderate- or high-risk exposure, whereas 227 were low risk. The difference between the date of symptom onset and the date of swab collection was a median of 6 days and a mean of 5 days for symptomatic participants. Most of those tested were female (216, 67%) and aged between 30 and 39 years (140, 43%). Eight of 324 (2%) tested positive via rRT-PCR for SARS-CoV-2 infection. Most of the HCWs tested were nurses (203, 63%), followed by nursing aides (61, 19%), medical doctors (37, 11%), laboratory personnel (12, 4%), and radiology technicians (6, 2%).

Four (50%) of the COVID-19–positive HCWs belonged to the 30- to 39-year age-group. Six were female (75%). Four were nurses (50%), three were laboratory personnel (38%), and one was a medical doctor (13%). One had asthma (13%), diabetes (13%), hypertension (13%), and dyslipidemia (13%). Another one had obesity (13%). Six had no comorbidities (75%).

Of the eight positives, only one, a 31/F nurse, was managing patients on a COVID-19 ward. She was classified to have low-risk exposure because of proper PPE use during interactions with patients. Seven others belonged to two different hospital departments. The first group consisted of a 48/M infectious disease physician and his nurses, a 32/F and a 30/M, who both lived in the same apartment during the community quarantine period. All three of them were part of a clinical team managing tuberculosis (TB) patients. These patients subsequently tested negative for SARS-CoV-2 after screening.^9^ The said physician who first developed COVID-19–like signs and symptoms had low-risk exposure to COVID-19 patients. The nurses, however, were classified to have high-risk unprotected exposure to the doctor, who was eventually confirmed COVID-19 positive. Seventeen days after the last COVID-19–positive HCW from the TB ward was detected, another nurse, 32/F, tested positive.

The second group consisted of medical technologists (24/F, 23/F, and 42/F), who lived together during the community quarantine period in Manila and were working in SLH’s HIV clinic. They were initially classified to have low-risk exposure with symptoms. However, during contact tracing, it was discovered that one of their housemates developed symptoms a few days before they were screened.

Seven of eight of the positive HCWs presented with respiratory symptoms such as cough (5), sore throat (4), rhinorrhea (3), and shortness of breath (1). Non-respiratory symptoms included loss of taste (3), loss of smell (3), loss of appetite (2), fever (1), vomiting (1), headache (5), nausea (1), chills (1), joint ache (1), and muscle ache (1)*.* Other symptoms reported were diarrhea (1) and fatigue (1). Two reported no symptoms. Of all these symptoms, only loss of smell (*P* = 0.004), loss of taste (*P* = 0.003), and loss of appetite (*P* = 0.02) were associated with confirmed COVID-19.

## DISCUSSION

Among the 324 HCWs we screened for COVID-19, eight were positive, and only one had worked in a COVID-19 ward. The number of COVID-19 cases among HCWs in this study represents a small number compared with the Philippines’ total of 2,710 HCWs.^2^ Although the number of COVID-19–positive HCWs in our study is small, some comparison with other studies is possible. In a study of HCWs in a tertiary hospital in Wuhan, China, 72% of 110 COVID-19–positive HCWs were females, with a median age of 36.5 years.^10^ This is similar to our study where six of eight of the COVID-19–positive HCWs were females and four of eight belonged to the 30- to 39-year age-group. The same study reported that 56% and 24% of the affected HCWs were nurses and physicians, respectively, and 66% of the COVID-19–positive HCWs were not tending to COVID-19 patients.^10^ In our screening, half of the positive hospital staff were nurses, followed by laboratory personnel, and only one was a physician. All except one of the positive HCWs were assigned to non–COVID-19 areas. Further analysis such as sequencing of the SARS-CoV-2 viromes from the affected HCWs could be pursued in the future to investigate epidemiological links among COVID-19–positive individuals.

Another study from a hospital in Wuhan reported that myalgia (100%), fever (86%), dry cough (71%), diarrhea (64%), headache (57%), and pharyngalgia (50%) were the most common symptoms among 12 COVID-confirmed HCWs.^11^ In our study, headache, cough, and sore throat were also among the most common. However, loss of taste, smell, and appetite were the only symptoms significantly associated with being COVID-19 positive.

The timely and effective infection prevention and control (IPC) measures of the hospital have potentially contained further transmission of COVID-19 to other susceptible HCWs, but these measures need to be sustained. Early preparations for COVID-19 were prompted by the arrival of the first two cases in SLH in late January, over a month before community transmission was announced in Manila in March. By February, a COVID-19 triage was set up at the entrance of the hospital to screen for COVID-19 signs and symptoms among patients arriving at the hospital. This allowed each patient to be managed and isolated, if needed, while the HCWs wore proper PPE.

A study of affected HCWs from a university hospital in Wuhan, China, observed a large difference in hospital IPC before and after the outbreak.^12^ Before the outbreak, only 78% of HCWs strictly followed hand hygiene, 53% strictly followed proper donning and doffing of PPE, 66% always wore masks, and 52% wore gloves in routine work. Seventy-seven percent of the hospital staff believed that the lack of appropriate PPE caused their infection.^12^

In SLH, before assignment of HCWs to COVID-19 areas, knowledge on the proper use of PPE was ensured by reorienting them on already standard hospital practices on donning and doffing. Despite challenges with resources in the months after March, the hospital was able to conserve their PPEs by implementing modified hospital guidelines on PPE use. This allowed the staff in non–COVID-19 areas to use less PPEs depending on the level of risk in the hospital areas they were assigned to. For example, in the COVID triage area, nurses who screen patients only wear a face mask and goggles/face shield, and doctors who assess patients only wear a disposable gown, N95 mask, and gloves.

Another study from Wuhan reported that psychosocial or emotional stressors of HCWs during the time of COVID-19 included disease-related issues (81%), worry about families’ health, and negative Internet news (40%).^12^ At SLH, HCWs were provided with regular briefing and de-briefing sessions with the hospital psychologist for mental health support. This was designed to address their worries and anxieties, particularly those stemming from occupational exposure to COVID-19.

San Lazaro Hospital’s patient safety office and the administrators of each department ensured regular assessment among HCWs for breaches in IPC protocols while being exposed to COVID-19 patients. Hospital staff were also routinely evaluated for the development of respiratory signs and symptoms. Those who needed medical attention were referred to the employees’ medical clinic.

## CONCLUSION

Early initiation of COVID-19 screening for HCWs and the preparation for the outbreak may have reduced the risk of infection in SLH. These practices allowed SLH to continue to provide hospital care for their patients. Healthcare workers are not only at risk of contracting COVID-19 from patients but also pose risks to immunocompromised patients if they are infected. To prevent and control COVID-19 transmission, HCWs should be closely monitored and examined routinely in an accessible clinic dedicated for hospital staff. Screening for SARS-CoV-2 infection should also be made available for them and should be performed on a regular basis. The reported seven cases of COVID-19 identified among HCWs in the course of the epidemic highlight the importance of such recommendations. In monitoring HCWs, we protect one of our most valuable assets against COVID-19.

## Supplemental table

Supplemental materials
