# Supplementary material for: COVID-19 Screening for Healthcare Workers in a Tertiary Infectious Diseases Referral Hospital in Manila, the Philippines
Source: Am J Trop Med Hyg. 2020 Jul 29;103(3):1211–4. doi: 10.4269/ajtmh.20-0715 (PMC7470521; doi:10.4269/ajtmh.20-0715)
Supplement: Supplementary file 1 [file tpmd200715.SD1.docx]

Supplementary table

**Table 1. Characteristics of 324healthcare workers from San Lazaro Hospital screened for COVID-19**

| **Characteristics** |  | **All** | **Covid-19** | **Non-Covid** | **p-value** |
| --- | --- | --- | --- | --- | --- |
| **Overall** |  | 324 | 8 | 316 |  |
| **Age (years)** | N | 324 | 8 | 316 |  |
|  | mean (SD) | 36 (9) | 33 (8) | 36 (9) |  |
|  | median (range) | 32 (23, 63) | 31.5 (23, 48) | 32 (23, 63) | 0.39 |
| **Age group (years)** | | | | | |
|  | 20-29 | 88 (27) | 2 (25) | 86 (27) |  |
|  | 30-39 | 140 (43) | 4 (50) | 136 (43) |  |
|  | 40-49 | 68 (21) | 2 (25) | 66 (21) |  |
|  | 50-59 | 24 (7) |  | 24 (8) |  |
|  | 60-69 | 4 (1) |  | 4 (1) |  |
| **Sex** | | | | | |
|  | Female | 216 (67) | 6 (75) | 210 (66) | 0.72 |
|  | Male | 108 (33) | 2 (25) | 106 (34) |  |
| **Occupation** | | | | | |
|  | Nurse | 203 (63) | 4 (50) | 199 (63) |  |
|  | Medical doctor | 37 (11) | 1 (13) | 36 (11) |  |
|  | Nursing aide | 61 (19) |  | 61 (19) |  |
|  | Radiology technician | 6 (2) |  | 6 (2) |  |
|  | Laboratory personnel | 12 (4) | 3 (38) | 9 (2) |  |
|  | Clerk | 4 (1) |  | 4 (1) |  |
|  | Other HCW | 1 |  | 1 |  |
| **Level of Exposure** | | | | | |
|  | Low Risk | 227 (70) | 4 (50) | 223 (71) | 0.25 |
|  | High Risk | 97 (30) | 4 (50) | 93 (29) |  |
| **Signs and symptoms** | | | | | |
| **Fever** | No | 320 (99) | 7 (88) | 313 (99) |  |
|  | Yes | 4 (1) | 1 (12) | 3 (1) | 0.1 |
| **Cough** | No | 157 (48) | 3 (38) | 154 (49) | 0.76 |
|  | Yes | 161 (50) | 5 (62) | 156 (49) |  |
| **Sore throat** | No | 100 (31) | 4 (50) | 96 (30) | 0.37 |
|  | Yes | 217 (67) | 4 (50) | 213 (67) |  |
| **Running nose** | No | 191 (59) | 5 (62) | 186 (59) | 1 |
|  | Yes | 126 (39) | 3 (38) | 123 (39) |  |
| **Shortness of breath** | No | 287 (89) | 8 (100) | 279 (88) | 1 |
|  | Yes | 28 (9) |  | 28 (9) | 1 |
| **Loss of smell** | No | 241 (74) | 4 (50) | 237 (75) | 0.004 |
|  | Yes | 13 (4) | 3 (38) | 10 (3) |  |
| **Loss of taste** | No | 241 (74) | 4 (50) | 237 (75) | 0.003 |
|  | Yes | 12 (4) | 3 (38) | 9 (3) |  |
| **Conjunctivitis** | No | 320 (99) | 8 (100) | 312 (99) | 1 |
|  | Yes | 4 (1) |  | 4 (1) |  |
| **Diarrhea** | No | 282 (87) | 7 (88) | 275 (87) | 1 |
|  | Yes | 42 (13) | 1 (12) | 41 (13) |  |
| **Fatigue** | No | 269 (83) | 7 (88) | 262 (83) | 1 |
|  | Yes | 55 (17) | 1 (12) | 54 (17) |  |
| **Malaise** | No | 312 (96) | 8 (100) | 304 (96) | 1 |
|  | Yes | 12 (4) |  | 12 (4) |  |
| **Headache** | No | 185 (57) | 3 (38) | 182 (58) | 0.3 |
|  | Yes | 139 (43) | 5 (62) | 134 (42) |  |
| **Joint pains** | No | 291 (90) | 7 (88) | 284 (90) | 0.52 |
|  | Yes | 33 (10) | 1 (12) | 32 (10) |  |
| **Loss of appetite** | No | 314 (97) | 6 (75) | 308 (97) | 0.02 |
|  | Yes | 10 (3) | 2 (25) | 8 (3) |  |
| **Muscle pain** | No | 245 (76) | 7 (88) | 238 (75) | 0.69 |
|  | Yes | 79 (24) | 1 (12) | 78 (25) |  |
| **Epistaxis** | No | 320 (99) | 8 (100) | 312 (99) | 1 |
|  | Yes | 4 (1) |  | 4 (1) |  |
| **Rash** | No | 320 (99) | 8 (100) | 312 (99) | 1 |
|  | Yes | 4 (1) |  | 4 (1) |  |
| **Vomiting** | No | 319 (98) | 7 (88) | 312 (99) | 0.12 |
|  | Yes | 5 (2) | 1 (12) | 4 (1) |  |
| **Chills** | No | 309 (95) | 7 (88) | 302 (96) | 0.32 |
|  | Yes | 15 (5) | 1 (12) | 14 (4) |  |
| **Nausea** | No | 310 | 7 (87.5) | 303 (96) | 0.3 |
|  | Yes | 14 | 1 (12.5) | 13 (4) |  |
| **Comorbidities** |  |  |  |  |  |
| **Asthma** | No | 301 (93) | 7 (88) | 294 (93) | 0.449 |
|  | Yes | 23 (7) | 1 (12) | 22 (7) |  |
| **Cancer** | No | 322 (99) | 8 (100) | 314 (99) | 1 |
|  | Yes | 2 (1) |  | 2 (1) |  |
| **Chronic Kidney Disease** | No | 321 (99) | 8 (100) | 313 (99) | 1 |
|  | Yes | 3 (1) |  | 3 (1) |  |
| **Chronic Liver Disease** | No | 323 (100) | 8 (100) | 315 (100) | 1 |
|  | Yes | 1 (0) |  | 1 (0) |  |
| **Diabetes** | No | 298 (92) | 7 (88) | 291 (92) | 0.492 |
|  | Yes | 26 (8) | 1 (12) | 25 (8) |  |
| **Heart disease** | No | 316 (98) | 8 (100) | 308 (97) | 1 |
|  | Yes | 8 (2) |  | 8 (3) |  |
| **Hypertension** | No | 257 (79) | 7 (88) | 250 (79) | 1 |
|  | Yes | 67 (21) | 1 (12) | 66 (21) |  |
| **Obesity** | No | 267 (82) | 7 (88) | 260 (82) | 1 |
|  | Yes | 57 (18) | 1 (12) | 56 (18) |  |
| **Dyslipidemia** | No | 323 (100) | 7 (88) | 316 (100) | 0.025 |
|  | Yes | 1 (0) | 1 (12) |  |  |
| **At least one underlying disease** | No | 5 (2) |  | 5 (2) | 1 |
|  | Yes | 319 (98) | 8 (100) | 311 (98) |  |
| **Duration between onset of symptoms and swab collection (within 14 days)** | N | 244 | 7 | 237 | 0.298 |
|  | mean (SD) | 6 (3) | 7 (3) | 6 (3) |  |
|  | median (range) | 6 (0, 14) | 8 (0, 14) | 5 (0, 14) |  |
